# 2D photos are great, but what about 3D imaging?

**DOI:** 10.1007/s00417-021-05479-4

**Published:** 2021-10-30

**Authors:** Senmao Li, Alexander C. Rokohl, Yongwei Guo, Ludwig M. Heindl

**Affiliations:** 1 Department of Ophthalmology, University of Cologne, Faculty of Medicine and, University Hospital of Cologne, Kerpener Strasse 62, 50937 Cologne, Germany; 2 Eye Center, Second Affiliated Hospital, School of Medicine, Zhejiang University, Hangzhou, China

Dear Editor,

With great interest, we read the article “Analysis of surgical outcome after upper eyelid surgery by computer vision algorithm using face and facial landmark detection” by our highly appreciated colleagues İlke Bahçeci Şimşek and Can Şirolu [1].

The authors provided us some very important insights into a computer vision algorithm using the face and facial landmark detection system for normalizing and calibrating photographs. This system offers a simple, standardized, objective, and reproducible assessment method for patients who underwent upper eyelid blepharoplasty. We fully agree that this is a first step of using artificial intelligence for evaluating the outcome of blepharoplasty in a more standardized fashion. However, there are some shortcomings in this study that might be addressed.

In this study [1], the original photos were captured by a Nikon D90 SLR (Single Lens Reflex) digital camera. Therefore, all the measurements were based on two-dimensional (2D) photos. Traditionally, craniofacial anthropometry is mainly obtained by direct caliper measurements or 2D photogrammetry. However, direct anthropometry using a caliper is not only time-consuming but also extremely dependent on patient’s compliance. Nowadays, informed patients are increasingly aware that these technologies cannot exactly acquire and quantify the complicated three-dimensional (3D) morphology of human faces in a standardized manner [2]. In addition, 2D photogrammetry falls short of proper 3D facial depth, and inaccuracy has been reported [2]. Three-dimensional imaging not only presents, in particular, the periocular region more vividly and realistically to both the surgeon and the patient but is also significantly more accurate compared to 2D images due to the use of the 3rd dimension.

**Fig. 1:**
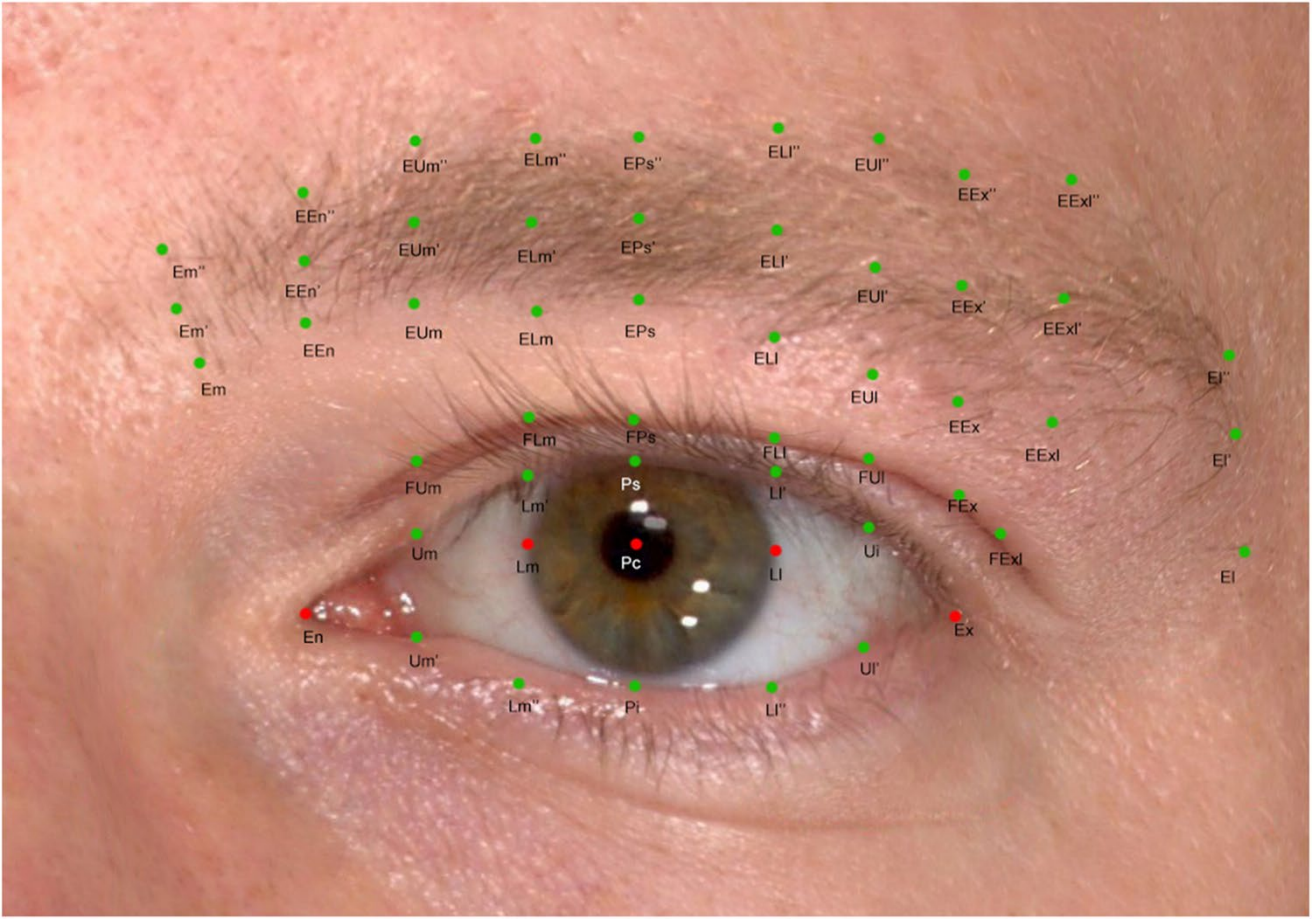
Fifty-two three-dimensional anthropometric landmarks of the periocular region elucidated in a two-dimensional modality. Five prime points are first located on 3D surface models, including the endocanthion, exocanthion, pupillary center, as well as the medial and lateral corneoscleral limbus (horizontal to the pupillary center)

In recent decades, novel non-invasive 3D imaging technologies are replacing both classic direct anthropometry (using rulers and calipers) and 2D digital photogrammetry. Three-dimensional imaging technologies allow operators to assess facial or mammary morphology and their alterations over time or after treatment, as well as differences between genders, ages, or ethnicities by analyzing the highly detailed metrical measurements on 3D surfaces. According to our studies, stereophotogrammetry and our landmark location protocol (Fig. 1) for the periocular region yielded very good reliability for a series of 2D and 3D liner, curvilinear, and angular measurements [2]. Furthermore, this 3D imaging system and landmark protocol was applied on describing the periocular region morphology and assessing its relationship with aesthetics in a European population [3]. In addition, we have applied this 3D imaging system for medial canthal tendon laxity and lower eyelid tension [4, 5].

In summary, more precise and vivid results could be achieved by using 3D imaging with a validated landmark protocol, instead of simple 2D photos. In addition, it would be interesting to combine 3D imaging and a computer vision algorithm with a periocular landmark detection system for improving the accuracy as well as provide patients best care using state-of-the-art technologies including artificial intelligence.
